# Zebrafish Sensitivity to Botulinum Neurotoxins

**DOI:** 10.3390/toxins8050132

**Published:** 2016-05-03

**Authors:** Kamalakar Chatla, Patricia S. Gaunt, Lora Petrie-Hanson, Lorelei Ford, Larry A. Hanson

**Affiliations:** 1 Department of Basic Science, College of Veterinary Medicine, Mississippi State University, Mississippi State, MS 39762, USA; chatla.kamalakar@gmail.com (K.C.); lora@cvm.msstate.edu (L.P.-H.); ford@cvm.msstate.edu (L.F.); 2 Thad Cochran National Warmwater Aquaculture Center, College of Veterinary Medicine, Mississippi State University, Stoneville, MS 38756, USA; gaunt@cvm.msstate.edu

**Keywords:** botulinum neurotoxins, zebrafish, lethal dose 50

## Abstract

Botulinum neurotoxins (BoNT) are the most potent known toxins. The mouse LD_50_ assay is the gold standard for testing BoNT potency, but is not sensitive enough to detect the extremely low levels of neurotoxin that may be present in the serum of sensitive animal species that are showing the effects of BoNT toxicity, such as channel catfish affected by visceral toxicosis of catfish. Since zebrafish are an important animal model for diverse biomedical and basic research, they are readily available and have defined genetic lines that facilitate reproducibility. This makes them attractive for use as an alternative bioassay organism. The utility of zebrafish as a bioassay model organism for BoNT was investigated. The 96 h median immobilizing doses of BoNT/A, BoNT/C, BoNT/E, and BoNT/F for adult male Tübingen strain zebrafish (0.32 g mean weight) at 25 °C were 16.31, 124.6, 4.7, and 0.61 picograms (pg)/fish, respectively. These findings support the use of the zebrafish-based bioassays for evaluating the presence of BoNT/A, BoNT/E, and BoNT/F. Evaluating the basis of the relatively high resistance of zebrafish to BoNT/C and the extreme sensitivity to BoNT/F may reveal unique functional patterns to the action of these neurotoxins.

## 1. Introduction

*Clostridium botulinum* is a Gram-positive, spore-forming, anaerobic, and rod-shaped bacterium that produces extremely potent neurotoxins. Their ability to effect neuromuscular paralysis has made them useful for treatment of muscle hyperactivity, blepharospasm, strabismus, and cosmetic defects [1]. Botulinum neurotoxins (BoNTs) are considered high threats for use as bioterrorism or biological warfare agents, because minute quantities can quickly cause neuro-paralytic illness, which terminates in respiratory failure [2]. *Clostridium botulinum* contamination of food and colonization of wounds and the gastrointestinal tract are the most common causes of botulism toxicity.

Botulism is seen in horses, cattle, birds, and fish, with dairy cattle, horses, and some species of fish being more sensitive to BoNT/E than mice [3,4,5,6,7]. Mouse bioassays are traditionally used to test the activity or concentration of BoNTs. There are several other diagnostic methods available to detect the causative agents of botulism outbreaks, such as polymerase chain reaction (PCR) for *C. botulinum*, anaerobic bacterial culture, and the enzyme-linked immunosorbent assay (ELISA). These assays, including the mouse bioassay, failed to detect BoNT/E in sera of catfish affected with visceral toxicosis of catfish (VTC) [3]. Possible reasons these assays did not work include the presence of the toxin in the affected fish in the absence of the *C. botulinum* organism (in the case of the PCR assay), the presence of low toxin levels, and variation in sensitivity to BoNTs in different animals [3,8]. Modeled after the mouse bioassay, a catfish serum neutralization assay was developed, which detected the presence of BoNT/E in catfish with VTC. This assay was confirmed by endopep mass spectrometry. In channel catfish aquaculture, VTC is a sporadic disease caused by BoNT/E. Clinical signs of VTC are erratic swimming and progressive muscular weakness leading to paralysis, lethargy, and death. Commonly-observed internal lesions include chylous or clear fluid (ascites) in the coelomic cavity, congested spleen, intussusception of the intestinal tract, and eversion of the stomach into the oral cavity [3].

During production of purified BoNTs for the pharmaceutical industry, the potency must be determined prior to marketing using the traditional standardized mouse LD_50_ assay. It is unknown how many mice are used for this assay, but in 2010, the estimated worldwide number used was 600,000 per year [9].With the increasing use of BoNTs in the treatment of neuromuscular diseases, pain relief and for cosmetic procedures [1], the current usage for BoNTs testing is likely much more. The use of mice is expensive, and bioassays on mammals for pharmaceutical production is controversial especially when using death as an endpoint [9].

The zebrafish is an oviparous cyprinid with high fecundity and exhibits physiological and morphological similarities to other vertebrates. Moreover, the zebrafish is an attractive and widely-used model organism for developmental biology, biomedical, immunological, genetic, and toxicology studies [10,11,12,13,14,15,16]. Zebrafish have been used to study the effects of cholera toxin and *Clostridium difficile* toxin [10,11,12,13,14,15,16]. There are many advantages of using the zebrafish as a model organism for BoNT studies. The zebrafish genome is fully sequenced; various recombinant zebrafish are available, as are polyclonal and monoclonal antibodies to cellular markers. Additional advantages of using zebrafish in BoNT research are the ability to produce large numbers of offspring in a short time; faster development compared to other vertebrates, low maintenance, and small space requirements for a large number of animals. We previously reported the utility of zebrafish to detect VTC and their sensitivity to BoNT/E as an alternative to the use of small channel catfish [17]. That study compared the sensitivity of zebrafish to channel catfish fingerlings and was evaluated using neutralizing antibodies to confirm the serotype of the toxin. Additionally, we used death as an endpoint and did not establish 50% endpoint dose effects. The object of this study was to evaluate the utility of zebrafish as a bioassay model for other BoNT serotypes and to evaluate the use of an endpoint that allows the subject to be euthanatized before lethal BoNT toxicosis. Therefore, we evaluated the median dose of BoNT serotypes A, C, E, and F that caused immobility in the zebrafish and refer to this as the median immobility dose (ID_50_).

## 2. Results

### 2.1. BoNT/A 96 h Median Immobilizing Dose

**Figure 1:**
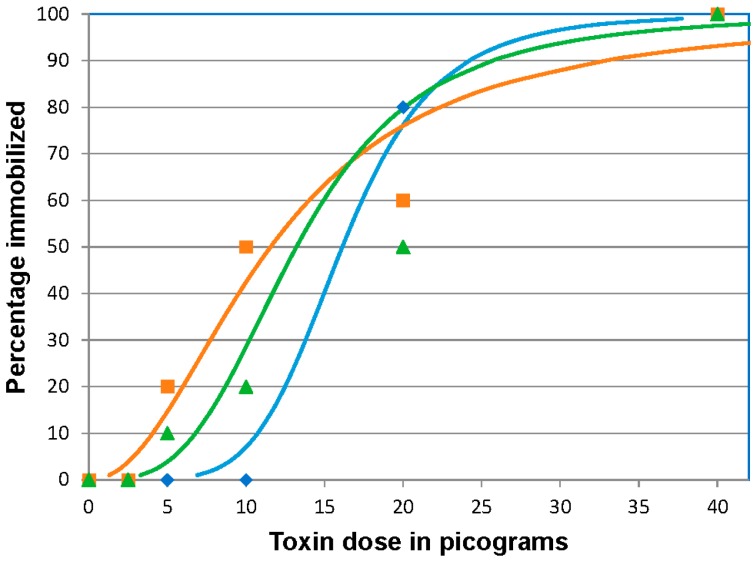
Botulinum neurotoxin A (BoNT/A) 96 h immobilizing dose response curves for zebrafish. Fish were injected intracoelomically with 10 µL of 40, 20, 10, 5, or 2.5 pg/fish BoNT/A diluted in gelatin phosphate buffer, (*n* = 10 fish/treatment/replicate). The results of three replicates are shown. The curves indicate the calculated dose effect by logit analysis on each replicate. The actual percentage immobilized for each dosage in each replicate is indicated by the symbol corresponding to the calculated curve of the same color. Blue curve and diamonds, orange curve and squares and green curve and triangles represent data from replicates 1, 2 and 3 respectively.

In all three replicates, 100% immobilization was observed at the highest dose (40 pg BoNT/A /fish). At 20 picograms (pg)/fish, the majority of fish (22/30) became immobilized, but all remaining fish exhibited typical muscular weakness (paresis) associated with botulism. At the doses of 10 and 5 pg/fish few fish reached the immobilized endpoint, but all fish rested at the bottom of the tank indicative of paresis. By the end of day 6 post-injection (pi), the fish were able to swim throughout the water column indicating some recovery but activity was lower than the control fish. At the 2.5 pg/fish dose and in the control group, none of the fish showed clinical signs of BoNT intoxication. Throughout the trials the control fish were actively swimming, schooling, and feeding. The ID_50_ values were 16.31 (95% CI 11.5 to 21.0), 11.56 (95% CI 7.6 to 17.0), and 13.21 (95% CI 9.4 to 18.6) pg/fish for the three separate challenges, respectively (Figure 1). The mean 96 h ID_50_ of BoNT/A, based on the three replicates was 13.7 ± 2.4 (SD) pg/fish providing an estimated coefficient of variation of 19%.

### 2.2. BoNT/C 96 h Median Immobilizing Dose

**Figure 2:**
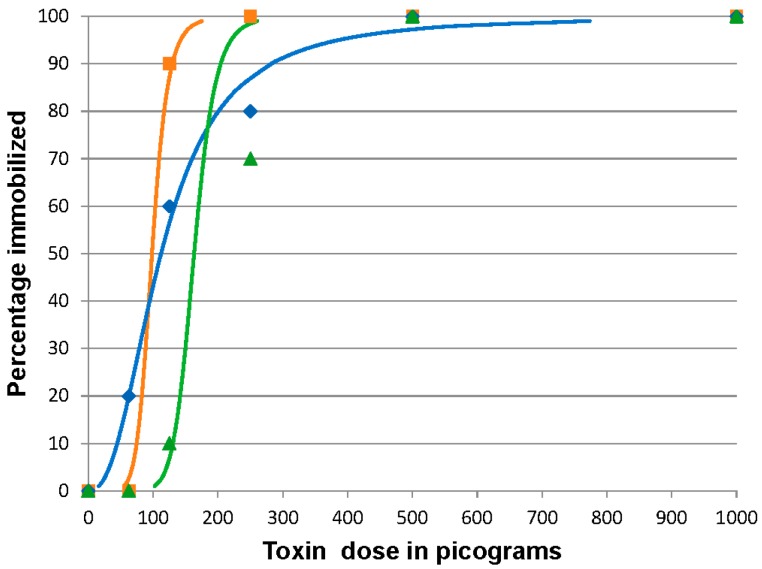
Botulinum neurotoxin C (BoNT/C) 96 h immobilizing dose response curves for zebrafish. Fish were injected intracoelomically with 10 µL containing 1000, 500, 250, 125, or 62.5 pg BoNT/C in gelatin phosphate buffer (*n* = 10 fish/treatment/replicate). The results of three replicates are shown. The curves indicate the calculated dose effect by logit analysis on each replicate. The actual percentage immobilized for each dosage in each replicate is indicated by the symbol corresponding to the calculated curve of the same color. Orange curve and squares, blue curve and diamonds, and green curve and triangles represent data from replicates 1, 2 and 3 respectively.

In the three replicates, 100% of the fish became immobilized at the highest doses, 1000 and 500 pg BoNT/C per fish. In the remaining doses, a range of 8–10, 1–9, and 0–2 per 10 fish/dose became immobilized in the 250, 125, and 62.5 pg/fish doses, respectively. The calculated ID_50_ values were 98.3 (95% CI 57.4 to 123.2), 112.2 (95% CI 67.2 to 163.6), and 163.3 pg/fish (95% CI 128.1 to 371) for three of the replicates, respectively (Figure 2). The mean of the combined values was 124.6 ± 34.2 pg/fish providing an estimated coefficient of variation of 30%. Since this dose was ten-fold higher than expected, we tested the potency of the preparation by intraperitoneally (IP) injecting mice with 100 pg of the BoNT/C. The BoNT/C-injected mice succumbed to the toxin by 24 h pi. Fish that were affected by the toxin but did not become immobilized showed no substantial recovery by day 7 post injection.

### 2.3. BoNT/E 96 h Median Immobilizing Dose

**Figure 3:**
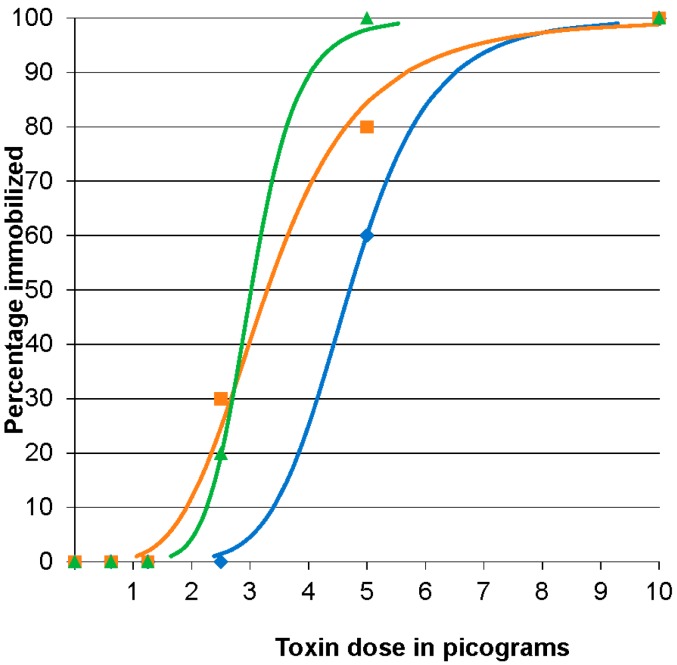
Botulinum neurotoxin E (BoNT/E) 96 h immobilizing dose response curves for zebrafish. Fish were injected intracoelomically with 10 µL containing 10, 5, 2.5, 1.25, or 0.62 pg BoNT/E activated with trypsin in gelatin phosphate buffer (*n* = 10 fish/treatment/replicate). The results of three replicates are shown. The actual percentage immobilized for each dosage in each replicate is indicated by the symbol corresponding to the calculated curve of the same color. Blue curve and diamonds, orange curve and squares and green curve and triangles represent data from replicates 1, 2 and 3 respectively.

In all three replicates, 100% of the fish were immobilized at the highest dose (10 pg BoNT/E per fish), at 5 pg/fish 60%, 80%, and 100% of the fish were immobilized, respectively, in three challenges. At 2.5 pg/fish, more than 50% of the fish showed clinical signs compatible with botulinum intoxication, but few reached the immobilized state at this dose. Most of these fish showed increased fin movement on day 6 compared to day 1 pi. In the 1.25 and 0.62 pg/fish and control doses, all of the fish appeared clinically normal and were actively swimming throughout the water column. The ID_50_ was 4.7 (95% CI 1.9 to 6.4), 3.29 (95% CI 2.4 to 4.5), and 3.01 pg/fish (95% CI 2.4 to 6) for the three replicates, respectively (Figure 3). The mean ID_50_ of BoNT/E in zebrafish was 3.7 ± 0.9 pg/fish providing an estimated coefficient of variation of 27%.

### 2.4. BoNT/F 96 h Median Immobilizing Dose

**Figure 4:**
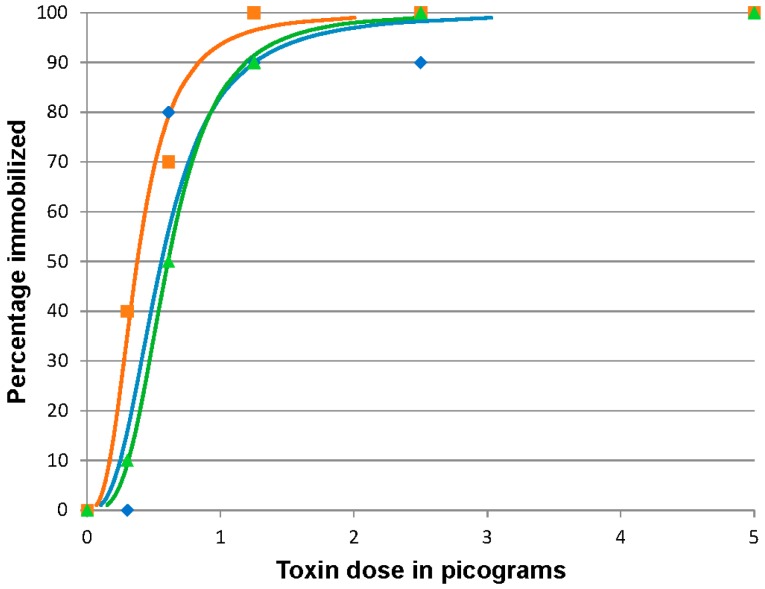
Botulinum neurotoxin F (BoNT/F) 96 h immobilizing dose response curves for zebrafish. Fish were injected intracoelomically with 10 µL containing 5, 2.5, 1.25, 0.62 or 0.3 pg BoNT/F diluted in gelatin phosphate buffer (*n* = 10 fish/treatment/replicate). The results of three replicates are shown. The actual percentage immobilized for each dosage in each replicate is indicated by the symbol corresponding to the calculated curve of the same color. Blue curve and diamonds, orange curve and squares and green curve and triangles represent data from replicates 1, 2 and 3 respectively.

Zebrafish were immobilized in all doses of injected BoNT/F. All BoNT/F injected fish exhibited clinical signs within 24 h pi, whereas control fish remained actively swimming, schooling, and feeding. By the end of day 4 pi, the affected zebrafish (that were not immobilized and removed) were again actively swimming. In the three replicates, the BoNT/F ID_50_ was 0.55 (95% CI 0.38 to 0.80), 0.38 (95% CI 0.19 to 0.54), and 0.61 (95% CI 0.41 to 0.85) pg/fish (Figure 4). The mean 96 h ID_50_ of BoNT/F in zebrafish was 0.51 ± 0.12 pg/fish providing an estimated coefficient of variation of 25%.

## 3. Discussion

This study demonstrates that BoNTs affect zebrafish in a similar fashion as other vertebrates. The mean ID_50_ values in 0.32 g male zebrafish were 16.31 pg of BoNT/A with characteristic clinical signs at 5 pg; 124.6 pg of BoNT/C with signs at 62.5 pg; 4.7 pg of BoNT/E with typical paralytic signs at 2.5 pg, and 0.61 pg/fish of BoNT/F with signs at 0.31 pg/fish. As a comparison, published LD_50_ values for 20 g mice are 24 pg for each of BoNT/A, BoNT/C, or BoNT/E, and 50 pg for BoNT/F [18]. Zebrafish showed clinical signs as early as 6 to 8 h pi when given the higher doses. At lower doses they showed signs by 24 to 48 h pi. We used the immobilization endpoint as an alternative to death because of animal welfare concerns. Immobilization is a result of flaccid paralysis and is much less stressful to animals than spastic paralysis, which is of much higher concern for animal welfare [19]. In our studies, we used five serial two-fold dilutions of toxins to determine the ID_50_; given the steep response curves, lower dilution ratios may have provided tighter distribution data. As it was, using three replicate experiments, we found that the estimated coefficient of variation in these assays ranged from 19% for BoNT/A to 30% for BoNT/C. This variation is higher than reported using mice in LD_50_ assays for BoNT/A, but precision can be improved through assay optimization. In our assays, we used a defined inbred strain of zebrafish; all were males and were age-matched. Other factors that may cause some variability in individual responses are variation in weight, nutritional status, and health status. Our assays could be optimized by using a tighter dilution series because the steep response patterns amplify small differences in only one to two critical dosages. These data support the use of zebrafish immobilization assays as an alternative to the mouse LD_50_ assay for both product analysis and for diagnostic work. Genetically-defined zebrafish are easily obtained and held in a laboratory environment. Variables that must be controlled in fish research that are not factors in mouse studies are the environmental temperature and water chemistry. In our study the temperature was maintained at a constant 25 °C, dissolved oxygen was maintained at saturation using aeration, and a flow-through water system was used to keep ammonia below detectable levels. Additional research is needed for specific applications, such as evaluating the effect of various matrices on the health of the fish. In a previous study, we found that the zebrafish bioassay, in conjunction with serum neutralization assays, was effective in detecting BoNT/E in channel catfish sera [17]. Alternatives to the mouse LD_50_ assay include the use of mouse hind leg paralysis [20] and mouse abdominal ptosis assays for BoNT/A [21]. Due to the small size of zebrafish, the use of localized paralysis assays would be more difficult and factors affecting persistence and recovery of localized paralytic assays would likely more closely model the therapeutic use of BoNTs.

In all vertebrates, clinical signs develop after the uptake of BoNT into peripheral nerves. In the cytoplasm of the peripheral nerves, the light chain (LC) of the BoNTs acts as a zinc-dependent endopeptidase and cleaves soluble N-ethymaleimide-sensitive factor protein receptor (SNARE) proteins, which leads to the blockage of acetylcholine release at the neuromuscular junction and synapses at post-ganglionic sympathetic and post-ganglionic parasympathetic nerve endings, and also at the autonomic ganglia [22,23]. Affected animals exhibited clinical signs based on the amount of toxin taken up by the nerves; in mice, signs were observed within 8 h pi at higher doses, and in lower doses the response was slower, sometimes longer than 24 h pi [3]. Zebrafish injected with doses of 2.5 pg of BoNT/E (13/30) and 5 pg of BoNT/A (23/30) showed clinical signs by 24 h pi. All immobilization was observed before 96 h pi. These observations indicate that neuron uptake of toxins and the response to the toxin is similar in zebrafish as it is in mice.

The persistence of muscle paralysis varies with the serotype of BoNT and the organism that is intoxicated [24]. The persistence of BoNT-induced paralysis depends on several factors: the ability and persistence of cleaved SNARE proteins to block the formation of functional SNARE complexes, the persistence of active BoNT LC in the cytosol, and also the alteration or recovery of the presynaptic terminal. Experimental studies showed that paralysis caused by BoNT/A is longer than BoNT/E in humans and rodents [24]. These toxins have been compared as examples of differential persistence [24]. Both cleave the SNARE protein, snaptosomal associated protein 25 kDa (SNAP-25). Two explanations for the longer persistence of BoNT/A in humans and rodents are: (1) The SNAP-25^A^ (SNAP-25 cleaved by BoNT/A) is integrated into a stable non-functioning SNARE complex where as SNAP-25^E^ is not; or (2) the enzymatically-active BoNT/A light chain persists longer in the cytoplasm than the BoNT/E light chain because it is unusually resistant to polyubiquitination, while BoNT/E is polyubiquitinated and subsequently degraded by the proteasome [24]. Our results, which are in agreement with other studies [1,9,20] suggest that recovery of BoNT intoxication depends on the dose; in lower doses, zebrafish showed progressive recovery with time. On day 7, lower-dose injected fish displayed minimal clinical signs of BoNT intoxication but still had visibly less activity compared to control fish. Previous studies have shown that the persistence of BoNT/F was of shorter duration compared to that of BoNT/A in mice [25,26]. Our experiments showed similar behavior in zebrafish; the fish injected with BoNT/F in lower doses such as 0.61 and 0.3 pg appeared to have quicker recovery compared to BoNT/A- and BoNT/E-injected fish. This faster recovery may be similar to the mechanisms discussed above: a more rapid incorporation of newly-synthesized vesicle-associated membrane protein (VAMP—the SNARE protein cleaved by BoNT/F) into functional SNARE complexes (compared to the persistent inhibitory action of SNAP-25^A^) or a more rapid degradation of BoNT/F LC.

Zebrafish were most resistant to BoNT/C and the ID_50_ was more variable compared to other BoNT serotypes tested. Previous studies in mammals also show high variability; the oral toxicity of BoNT/C for foxes varied from 10^3^ to 10^8^ times the mouse lethal dose (MLD), and the toxicity when IP administered in minks varied from 10^3^ to 10^5^ times MLD [27,28]. These studies suggest that BoNT/C toxicity is more variable within certain species than are the other BoNTs.

BoNT/C cleaves SNAP-25 and syntaxin proteins of the SNARE complex. The observed resistance of zebrafish might be due to the lack of crucial structural motifs, which aid BoNT/C binding to the syntaxin and SNAP-25. BoNT/C cleaves syntaxin 1b at Lys252-Ala253, only when they are inserted into the lipid bilayer [29]. BoNTs recognize their substrate via a double interaction, region A is the neurotoxin binding motif and region B contains the peptide bond to be cleaved [30]. There are several syntaxin isoforms expressed based on tissue and cellular distribution; syntaxin 1a and 1b are specifically expressed in nervous tissue [29]. To date, syntaxin 1a has not been identified in zebrafish. Even though the syntaxin 1b sequence is conserved in humans, mice, and cows; the zebrafish syntaxin has ten amino acid differences, five of these differences are downstream of the cleavage site (amino acid 253 to 288). SNAP-25 proteolysis depends on the presence Asn93 to Glu145 and Ile156 to Met202 regions [31]. These regions are conserved in humans, mice, and cows, but not in zebrafish. In the region Asn93 to Glu145, zebrafish have several amino acid differences and two and three gaps in SNAP-25A and SNAP-25B, respectively. Region Ile156 to Met202 also has a couple of amino acid differences, but no gaps. Since the BoNTs LC require proper binding before they can cleave their cognate substrate, the structural differences between the representative syntaxins and/or SNAP-25 of the mouse and the zebrafish might be the cause of zebrafish resistance to BoNT/C.

## 4. Conclusions

Our studies demonstrate the highly-sensitive and reproducible response of zebrafish to BoNT/A, BoNT/E, and BoNT/F. Additionally, the use of immobilizing dose endpoints is more humane than LD_50_ assays. These data suggest that the zebrafish ID_50_ assays would be useful bioassays for evaluation of these toxins. On the other hand, zebrafish were highly resistant to BoNT/C abrogating their use for screening this toxin. The differential sensitivity of zebrafish to BoNT/C compared to other serotypes and to other species of host animals combined with the availability of molecular tools and recombinant zebrafish would facilitate further research on mechanisms of resistance/sensitivity to this important toxin [14].

## 5. Experimental Section

### 5.1. Experimental Design

The ID_50_ experiments in this project were approved by the Mississippi State University Animal Care and Use Committee (Project #13-017 approved 5 March 2013) and the Institutional Biosafety Committee project (Project #014-09 approved 13 November 2009, modification approved 30 March 2010). For each experiment we used five two-fold serial diluted doses and a control (phosphate gelatin buffer—2 g of gelatin, 4 g of Na_2_HPO_4_ in 1 L dH20, pH 6.2) dose. In each experiment 10 zebrafish per dose were injected intracoelomically. BoNT was injected in the coelomic cavity posterior to the pelvic girdle with a 35 gauge needle attached to an insulin syringe. All fish were injected with 10 µL of an assigned toxin dose or control buffer and observed three times a day over a seven day period for clinical signs and lesions of BoNT intoxication, including abnormal swimming pattern, lack of tail movement, lethargy, paresis, exophthalmia, settling at the bottom of the tank, fin in-coordination, and erratic swimming. Fish that were quiescent at the bottom of the tank, unable to maintain an upright status, and unable to move their fins were considered immobilized. Immobilization was the endpoint for this assay. Immobilized fish were euthanized by immersion in water containing 300 mg/L tricaine methane sulfonate (MS-222, Tricaine-S, Western Chemical, Inc., Ferndale, WA, USA) [32]. After seven days, surviving fish were euthanized as above. Each experiment was repeated three times to evaluate reproducibility.

### 5.2. Zebrafish

All the experimental zebrafish (*Danio rerio*) were obtained from the Mississippi State University College of Veterinary Medicine (MSU-CVM) fish hatchery. These fish were propagated and nurtured in a specific pathogen-free environment according to standard operating procedures previously described [15,33]. Adult male zebrafish (bodyweight 0.32 ± 0.045 g mean ± SD) were used for experimental challenges. Experimentally-challenged zebrafish were transferred to 15 L aerated tanks receiving charcoal-filtered de-chlorinated municipal water at a rate of 0.5 L/min at 25 °C.

### 5.3. BoNTs Source and Handling

Purified BoNT/A, BoNT/C, BoNT/E, and BoNT/F holotoxins were purchased from Metabiologics, Inc (Metabiologics, Madison, WI, USA) [34]. BoNTs were handled in a Class II bio-safety cabinet equipped with HEPA filters (Contamination Control Inc., Landsdale, PA, USA). Each toxin was diluted to a stock concentration 10^4^ pg/uL with gel phosphate buffer. Each stock was further diluted to a working stock concentration. BoNT/A and BoNT/C were reduced by incubation with 20 mM dithiothreitol in gel phosphate buffer at room temperature for 30 min. BoNT/E was activated (nicked) with 5% trypsin (1:250 Difco, 0.05 g in 1 mL; 5 µL of 10^4^ pg/µL BoNT-E, 100 µL of 5% trypsin, 895 µL of gel phosphate buffer) at room temperature for 30 min prior to injection. The toxin or activated toxins were further diluted to nominal concentrations with gel phosphate buffer to deliver the following doses: BoNT/A: 40, 20, 10, 5, and 2.5 pg/fish; BoNT/C: 1000, 500, 250, 125, and 62.5 pg/fish; BoNT/E: 10, 5, 2.5, 1.25, and 0.61 pg/fish; and BoNT/F: 5, 2.5, 1.25, 0.61, and 0.3 pg/fish. Confirmation of BoNT/C activity was tested in mice using the same stock of BoNT/C for zebrafish injections. The median toxic dose of a mouse (100 pg) was IP injected into three Swiss-Webster male mice (18–20 g), and a control mouse was injected with gelatin phosphate buffer [35].

### 5.4. Statistical Methods

A logit model was used to determine the 96 h pi ID_50_ of challenged zebrafish, by taking the natural logarithm values of the dose concentration *vs.* the activity of the toxin using IBM SPSS-Statistics for Windows, Version 22.0 software (IBM Corporation. Armonk: New York, NY, USA, 2013) for regression analysis. The ID_50_ was calculated at 95% confidence intervals (CI). After day 4, no fish became immobilized in any of the challenges. Extending the duration of study did not increase the precision of LD_50_ in mice [20]. Therefore, our median immobilizing doses were determined from 96 h observation periods, but we continued to monitor the fish for clinical signs until day 7.
